# Local Coding Based Matching Kernel Method for Image Classification

**DOI:** 10.1371/journal.pone.0103575

**Published:** 2014-08-13

**Authors:** Yan Song, Ian Vince McLoughlin, Li-Rong Dai

**Affiliations:** National Engineering Laboratory of Speech and Language Information Processing, University of Science and Technology of China, Hefei, China; Beihang University, China

## Abstract

This paper mainly focuses on how to effectively and efficiently measure visual similarity for local feature based representation. Among existing methods, metrics based on Bag of Visual Word (BoV) techniques are efficient and conceptually simple, at the expense of effectiveness. By contrast, kernel based metrics are more effective, but at the cost of greater computational complexity and increased storage requirements. We show that a unified visual matching framework can be developed to encompass both BoV and kernel based metrics, in which local kernel plays an important role between feature pairs or between features and their reconstruction. Generally, local kernels are defined using Euclidean distance or its derivatives, based either explicitly or implicitly on an assumption of Gaussian noise. However, local features such as SIFT and HoG often follow a heavy-tailed distribution which tends to undermine the motivation behind Euclidean metrics. Motivated by recent advances in feature coding techniques, a novel efficient local coding based matching kernel (LCMK) method is proposed. This exploits the manifold structures in Hilbert space derived from local kernels. The proposed method combines advantages of both BoV and kernel based metrics, and achieves a linear computational complexity. This enables efficient and scalable visual matching to be performed on large scale image sets. To evaluate the effectiveness of the proposed LCMK method, we conduct extensive experiments with widely used benchmark datasets, including 15-Scenes, Caltech101/256, PASCAL VOC 2007 and 2011 datasets. Experimental results confirm the effectiveness of the relatively efficient LCMK method.

## Introduction

Visual matching is a core task of many content-based image retrieval and visual recognition applications. Existing visual matching algorithms generally comprise two closely related components: *visual content representation* and *similarity measurement* [1]. An image can conventionally be globally represented by low-level features such as GIST [2], Gabor filter, color or texture histograms computed over the entire image or over fixed regions. Using such methods, a convenient and compact representation can be achieved and used for visual similarity measurement [3], [4]. However a significant disadvantage is that global features can be sensitive to intra-category variations caused by different viewpoints, lighting conditions and background clutter. The consequence of this is degraded visual matching accuracy.

Local feature based representations have recently attracted much attention. For example, SIFT [5] and HoG [6], which are extracted from patches around detected interest points, or extracted in a dense grid over the image. Representing images using local feature sets is demonstrably more descriptive, discriminative and robust to intra-category variations compared to using a single global feature vector [7]. However representation by local feature sets in this fashion may be redundant, impacting the efficiency of the visual similarity measurement task. The problem is more challenging in that the feature sets have different cardinalities and are orderless.

The Bag-of-Visual Words (BoV) model, by far the most popular matching method to date, maps the local feature set into a fixed-length histogram. The process consists of two main phases: (i) *Feature quantization* assigns every local feature to the nearest visual words in a dictionary. The dictionary would generally have been obtained off-line through a clustering process on a large local feature set. (ii) *Spatial pooling* counts occurrences of visual words in the image (or in spatial regions) to form a histogram representation. BoV shares some advantages with global feature based representations. For example, visual similarity can be efficiently measured using a linear kernel on the histograms, or by using more accurate additive homogeneous kernels [8], [9]. However, quantization error (i.e. the difference between a local feature and its assigned visual word), is known to degrade the effectiveness [10]. Furthermore, the spatial context information of local features is ignored in BoV.

A plethora of extensions have built on the foundation of BoV. Many aim to reduce local feature quantization error, such as soft assignment coding [11] [12], local coding [13] [14] and sparse coding [15]. These use multiple visual words with locality or sparsity constraints to represent local features more accurately. Super-vector coding [16] and aggregated coding [17] approximate the Fisher vector [18] to achieve a better representation by exploiting first and/or second-order statistics from features in different image layouts. Further improvements in matching accuracy can be obtained by using image layout to introduce rough spatial correspondence between images, such as the spatial pyramid structures in [16], [19]. Spatial information could also be exploited to derive semantic mid-level features [20]–[24].

Apart from BoV, kernel based methods define visual similarity based on the set-level kernel, which is derived directly from the kernels in local feature space. Generally, this process [25], [26] first calculates local kernels over pairs of features, before aggregating the local kernels into set-level kernels. Parsana et al. [27] modifies the calculation of local kernels by integrating spatial information. Meanwhile, Boiman et al. [10] and Rematas et al. [28] proposed Nearest Neighbor (NN) classification techniques, called Naive Bayes Nearest Neighbor (NBNN), to classify images under the naive Bayes assumption. This employs the nearest image-to-class distance as a set-level kernel.

Although these methods are effective, many become impractical for large scale image sets due to the high computational and memory costs implicit in the calculation of local kernels. Several authors therefore use approximation techniques to reduce complexity. For example, NBNN uses a KD-tree implementation to approximate the nearest neighbor distance. Similarly, Efficient match kernels (EMK) [29] map local features to a low-dimensional feature space using constrained kernel singular value decomposition (CKSVD). Some other authors estimate a probabilistic distribution on sets of local features, and then derive similarity using distribution-based distance metrics [30]–[32].

In fact BoV-based and kernel-based methods are closely related. We will show in the next section how a local feature based visual matching framework can be derived to unify them both. From a local feature based visual matching perspective, we can see that the local kernel measuring the similarity between feature pairs, or between features and their reconstruction, plays an important role. Existing local kernels are mostly defined using Euclidean distance or its derivatives, based either explicitly or implicitly on a Gaussian noise assumption. However, such an assumption may not be valid for gradient based local features, e.g. SIFT and HoG, as has been demonstrated by several authors: For example, in [33] Jia et al. showed that the statistics of gradient based local features often follow a heavy-tailed distribution, which undermines the motivation for using Euclidean metrics. Similarly, Wu et al. [34] showed that a histogram intersection kernel (HIK) is more effective than Euclidean distance for supervised/unsupervised learning tasks with histogram feature. Meanwhile, second-order SIFT statistics with appropriate non-linearities were also shown to improve visual similarity measurement [35]. Some feature embedding methods have been shown to yield large performance improvements when used with linear SVM, such as square-root embedding [36], [37].

### Contributions

Motivated by recent progress in feature coding techniques [13]–[15], we develop a local coding based matching kernel (LCMK) method for efficient and effective visual matching. The proposed LCMK method shares the non-Euclidean assumption with [35], [36]. Yet a key difference is that we aim to learn an embedding function directly in the Hilbert space derived from a non-linear local kernel. Specifically, the method proposed in this paper has the following novel properties:

- We show that the existing BoV and kernel based methods can be unified using a more general local feature based visual matching, in which the effectiveness and efficiency of constructing a local kernel matrix is an important factor.
- Both BoV and kernel based methods can achieve efficiency by approximating an effective non-linear kernel, using a linear kernel with a non-linear embedding function. By contrast, we propose to learn the embedding function from the Hilbert space derived from the local kernel directly.
- The proposed LCMK method combines the advantages of both BoV and kernel based similarity measurements, yet will be shown to achieve a linear computational complexity. It is therefore an efficient and scalable method for measuring image level similarity.

The effectiveness of this method is demonstrated through image classification experiments on various datasets, including 15-Scenes, Caltech101/256 [19] [38] and PASCAL VOC 2007 and 2011 [39] [40]. The experimental results show superior performance compared to the state-of-the-art techniques based on SIFT features.

The rest of this paper is organized as follows. A general definition of local feature based visual matching is firstly introduced, following which, two main categories of similarity measurement are briefly reviewed and discussed. Next, the proposed method, to compute a compact image-level representation from local kernels, is presented in detail. This includes the analysis of its complexity in comparison with other methods. Finally, the experimental results are presented and analysed. The paper ends with a conclusion and discussion of potential future work.

## Methods

### Visual Matching

This section begins with a general definition of visual matching based local feature representation. Both BoV and kernel based methods are then reviewed from a visual matching point of view. Finally we discuss the relationship between these two methods in detail.

Specifically, assume that we are given two images 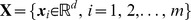, 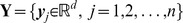, where 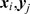 are *d*-dimensional local features extracted from the images. A generic image-level similarity measurement can be defined as
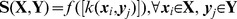
(1)where 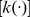 is the local kernel matrix over feature pair combinations of 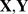, and 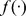 is the mapping function from local kernel matrix to set-level kernel.

For a collection of 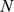 images 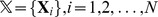, there are 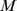 local features extracted 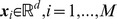, 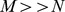. Visual matching can be stated as obtaining the image-level similarity matrix 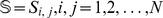 from local kernel matrix 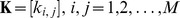. The time complexity of visual matching is 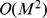, and the storage requirement for similarity kernel matrix 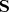 is 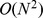, growing quadratically with the size of the image set. This leads to serious scalability problems.

#### BoV based Matching Methods

Given a dictionary of 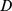 visual words 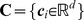, 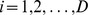, feature quantization approximates local feature 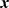 with its reconstruction
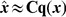
(2)where 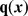 is the coefficient vector,
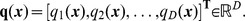



Generally, optimal 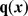 can be obtained by minimizing the quantization error 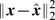. The simplest feature quantization uses hard-assignment coding, which encodes local features to their nearest visual word giving a coefficient vector 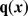 with one and only one nonzero entry. By contrast, soft-assignment coding represents local features as a linear combination of several visual words with respect to sparsity or locality constraints [15], [41].
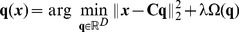
(3)where 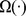 is a regularization term on the quantization coefficient vector. In sparse coding, the regularization term is in *L*_1_-norm form [15],
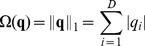



In local coding, an additional locality constraint is considered
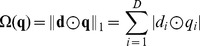
where 
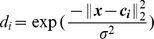
 is the distance from feature 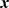 to visual word 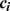, and 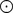 denotes element-wise product. In practice, a sum-to-one operator can be applied on quantization coefficient vector 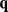 to achieve shift-invariance [15], [41].

After feature quantization, a pooling operator is generally needed to summarize the quantization coefficient vectors over a whole image or over large image regions. Generally, *L_p_*-norm operator 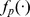 can be used [20], [42], [43],
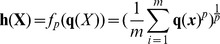
(4)where 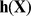 is a *D*-dimensional histogram vector. The parameter *p* is used to control the type of pooling operator: 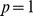,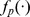 denotes average-pooling, and a convenient histogram representation is obtained; 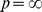 denotes max-pooling, which captures the most significant quantization coefficients in an image.

Finally, visual similarity between images 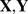 can be defined over image-level representation 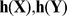 efficiently
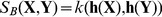
(5)



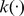
 is the kernel function measuring the visual similarity between BoV representations. Several popular kernel functions for image classification are listed below:

- Linear kernel: 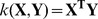

- Intersection kernel: 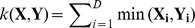

- Hellinger kernel: 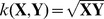

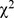
 kernel: 
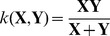

#### Kernel based Visual Matching

Given the local kernels 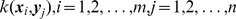 between two sets of features, a straightforward kernel based visual matching can be defined using
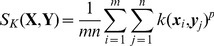
(6)where *p* is the exponent parameter to control the importance of the local kernel, with *p* = 1 equating to the sum match kernel in [25], and other values of *p* affecting the bias given to local kernels.

In NBNN [10], the image-to-class similarity is used instead of the image-to-image one, which can be formulated as
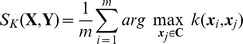
where 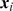 is the local feature in image 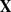, and 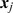 is the local feature in class 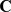.

Suppose there is a non-linear mapping 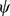 from feature space to a Hilbert space 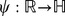, induced by local kernel 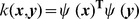. Eqn.6 can then be rewritten as
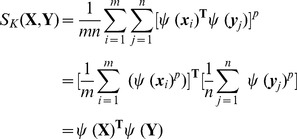
(7)


Despite their effectiveness, kernel based matching methods are generally computationally complex. Several approximations have thus been introduced to improve efficiency, such as PMK [32], EMK [29] *etc*.

#### Discussion

BoV based methods aggregate the local features into a single vector representation, which allows more efficient similarity measurement. The simplest way for aggregation is to average the feature vectors in an image. However, this may lose much information about underlying image content due to the diverse distribution characteristics of local features.

In BoV, a dictionary of visual words is trained off-line for partitioning the local feature space into Voronoi cells according to their distributions. In fact, these visual words act as a coordinate system. By mapping local features to the new coordinates during the feature quantization stage, pooling can be conducted on features in the same Voronoi cell without losing much information.

Kernel based methods aggregate the local kernels from different feature sets to derive a similarity measurement, which is close to our definition of visual matching in Eqn. (1). However, the computational complexity and storage requirement is too high for large scale image sets. A better way might be a combination of the advantages of both methods.

This is possible since both BoV and kernel based methods can be unified from a visual matching perspective. Specifically, from eqns. (4) and (5), BoV based similarity measures can be represented using
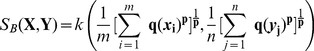
(8)which is similar to eqn. (7) with a linear kernel, by considering 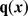 as a low-dimensional embedding of 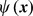.

It can be seen from eqn.(1) that the base kernel over a pair of local features plays an important role in matching based methods. However, existing methods generally use Euclidean distance to define a base kernel 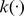, which may not be optimal for histogram based feature vectors, such as SIFT and HoG. To address this issue, previous methods generally find an explicit mapping function to approximate the non-linear kernel at either image or feature levels.

Unlike existing methods, we propose an efficient local coding based visual matching method, which aims to combine the strengths of both BoV and kernel based methods. The assumption is that local features for image classification, when densely extracted from the image, may exhibit intrinsic manifold structures. The soundness of this assumption has been supported by the success of recently proposed local coding methods [12], [13].

### Similarity Measurement via Proposed Efficient Local Coding based Matching Kernel

As mentioned, most local feature based matching methods are developed using Euclidean distance functions under the Gaussian noise assumption, probably for the sake of efficiency. However, local features, *e.g.* SIFT and HoG, generally follow a heavy-tailed distribution [21]. Euclidean based similarity measures may therefore yield a poor matching accuracy and have undesirable side-effects. Recent works, such as Laplacian sparse coding [44] and local coding [12], [13], demonstrate that improved matching accuracy is achievable by exploiting manifold structures during feature quantization.

In the following sections, we first describe the process of learning an embedding matrix from the Gaussian kernel, and then derive our proposed LCMK method for efficient visual matching, which aims to design a local kernel matrix that can incorporate neighborhood information for finding manifold structures in feature space. A low-dimensional embedding function is then learned by approximating this local kernel matrix.

#### Learning embedding from the Gaussian kernel

Suppose that we are given a set of randomly selected training features 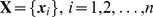. Let 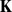 denote the kernel matrix defined on data set 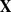. There is an implicit feature mapping from Euclidean space to a Hilbert space 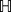, 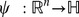, derived from a Gaussian kernel 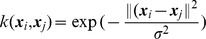. We aim to learn a *D*-dimensional projection 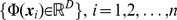 that can best approximate the original kernel matrix 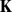.

Firstly, a set of *D* anchor points 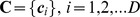 can be obtained by applying *k*-means clustering on data set 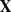. Let 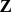 be the basis vectors 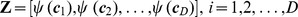, 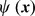 can then be approximated using
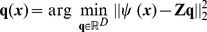
(9)where 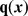 is a *D*-dimensional coefficient vector. Since eqn. (9) is convex quadratic, a closed-form solution can be found
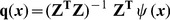
(10)


By replacing 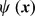 with 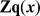, the original kernel function 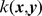 can be approximated as
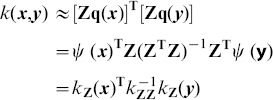
(11)where 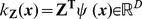, and 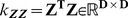. Since 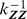 is positive definite, it can be decomposed as 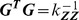 using Cholesky-decomposition. The local kernel can be further written as
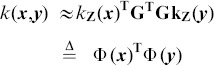
(12)where 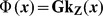 is a *D*-dimensional embedding of 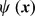.

Since local features generally follow a non-Gaussian distribution, it is beneficial to incorporate the neighborhood information in kernel matrices (*i.e.* 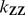 and 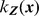) to exploit the latent manifold structures. An intuitive way is to add a locality constraint to eqn. (9).
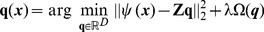
(13)where 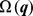 is the regularization term defined in eqn. (3). However, due to the non-convexity of eqn. (13), there is no closed-form solution. A computationally complex optimization procedure *e.g.* the feature sign algorithm [15], is generally required.

Another possible way is to use spectral analysis methods, such as Locality Preserving Projection (LPP) [45], Laplacian Eigenmap (LE) [46]. Given the data set 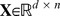, spectral analysis methods generally need to construct an un-direct graph represented by an 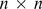 adjacency matrix 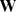, in which each non-zero entry 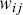 denotes the similarity between neighboring data. The spectral embedding matrix 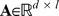 can constructed using eigenvectors 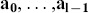, ordered according to their corresponding eigenvalues 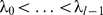, where eigenvector 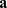 and eigenvalue 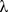 are obtained by solving the generalized eigen-decomposition problem as follows,
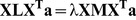
(14)where 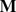 is a diagonal matrix with each entry 
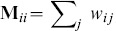
, and 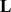 is a Laplacian matrix 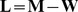. The computational complexity and storage requirement of constructing such graph is 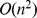, quadratic with the number of data points. For kernel based LPP, additional computation of the kernel matrix is needed. To address this issue, we propose to learn the embedding from the kernel matrix derived using the local coding technique.

#### Learning embedding from local coding based kernel

Our proposed algorithm for learning embedding matrix from the local coding based kernel is shown in Table 1. Given a set of *D* anchor points 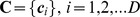, we propose to use following local coding of feature 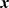, referring to the weight matrix construction step in LPP [45]

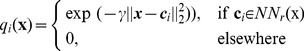
(15)where parameter 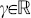 and 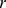 is the number of nearest anchor points. We found empirically that setting 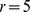 and 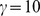 can achieve reasonable results. This local coding scheme is similar to the feature quantization in BoV method. The major difference is that in BoV, the quantization coefficient vectors are pooled together for image-level representation; whereas in LCMK, these vectors are used to approximate the kernel matrix 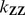 in eqn.(11). Let 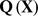 denote the quantization coefficient matrix of data set 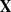, 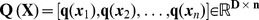.
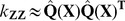
(16)where 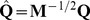 is the normalized coefficient matrix. 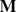 is the row sum of 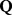. The local kernel between feature pair in eqn.(11) can then be defined as
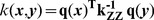
(17)where 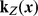 is replaced by the quantization coefficient vector 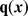. According to [47], there is a close relationship between 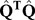 and 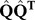. From the perspective of spectral analysis, the matrix 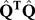 may be considered as an approximation of the weight matrix using the anchor points instead of the whole training set. The time complexity of constructing the coefficient matrix 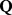 is 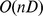, which scales linearly with 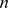 when the number of anchor points is fixed.

**Table:** **Table 1.** Algorithm 1: Learning embedding from local coding based matching kernel:LCMK.

Input: 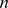 local features 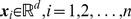 ; number of anchor points 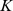 ;
Output: Embedding matrix 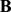
1. Generate 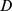 anchor points using k-means algorithm;
2. Obtain the adjacency matrix using local coding technique 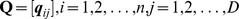 according to eqn.(15);
3. Calculate 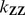 using eqn.(16);
4. Calculate the embedding matrix 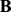 using 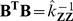

Since 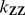 is positive definite, 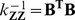. Eqn.(17) can then be simplified as
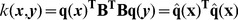
(18)where 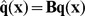.

#### Similarity measurement and complexity analysis

Given two images 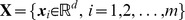, 
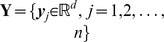
, the image-level similarity can be measured by substituting the local kernel in eqn.(7) with eqn.(18):
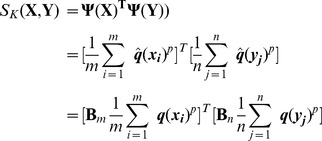
(19)where 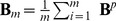. Since 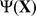 is finite and can be computed explicitly, we can first extract the image-level representation in a similar way to BoV, then apply the embedding on the image-image level representation.

Note that in practice, embedding matrix 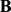 can be learned off-line, simultaneously with construction of anchor points. The time complexity of the proposed LCMK method mainly consists of (i) local coding of the features, and (ii) feature embedding and aggregating to form an image-level representation. Given a set of *n* features 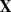 extracted from an image, the time complexity of local coding in eqn.(15) tends towards 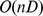, which scales linearly with *n* when the number of anchor points *D* is fixed. Furthermore, we use the efficient approximate *r*-nearest-neighboring algorithm and KD-tree implementation of [48] to reduce the computational complexity of feature embedding caused by a large number of anchor points. The time complexity of feature embedding and aggregating to image-level representation in eqn.(19) is basically 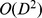. Overall, the computational cost of LCMK is much lower than that required to evaluate the matching kernel, which scales quadratically with *M*, the number of local features extracted from the whole image set, since 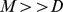. Compared to the BoV based visual similarity, the computational cost is slightly higher due to computation of embedding of the image representation. However, as will be seen in the following section, performance is much better.

## Experiments

To evaluate the effectiveness of the proposed LCMK method, we conduct extensive image classification experiments on 15-Scenes, Caltech101/256 and PASCAL VOC 2007/2011 datasets.

### Datasets

**Figure 1:**
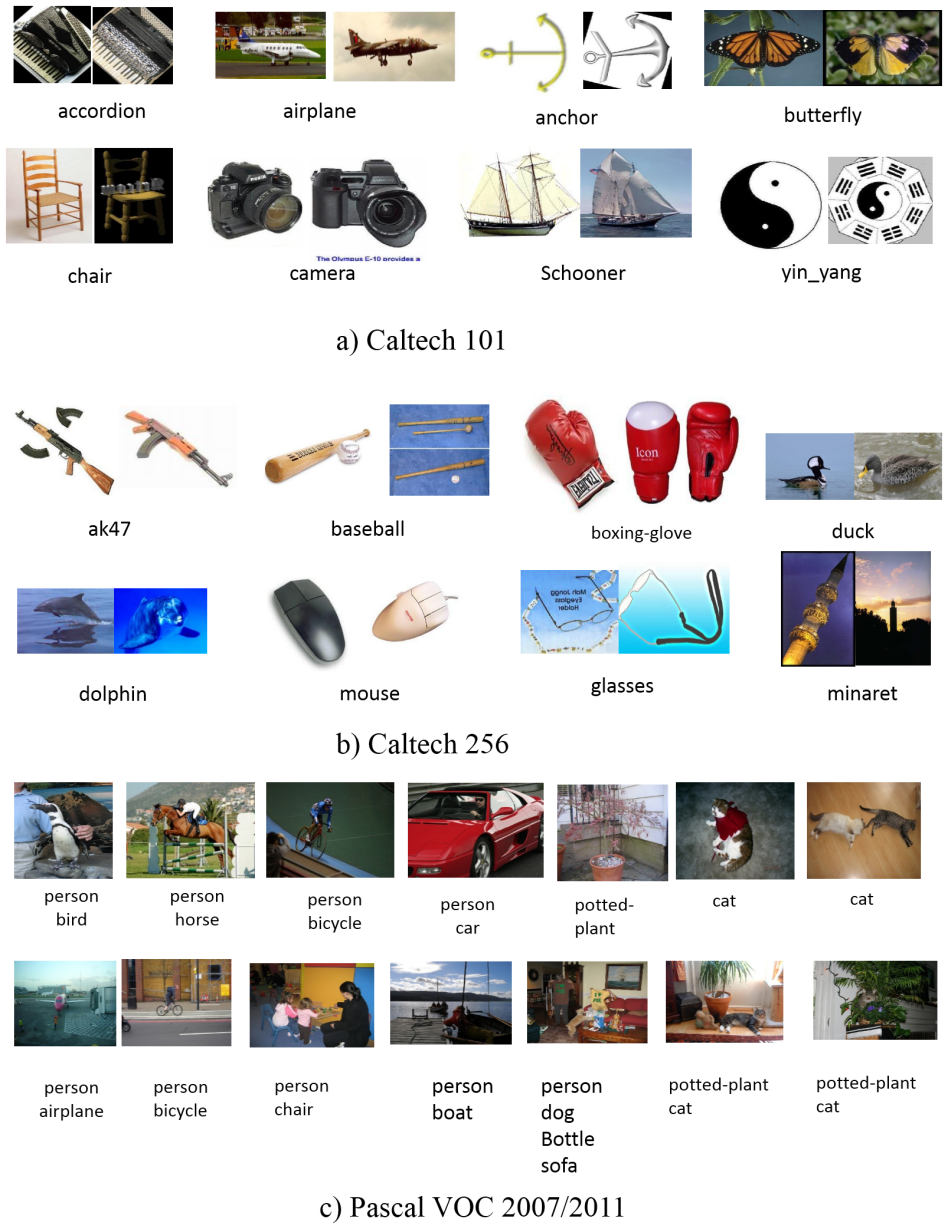
Some examples from Caltech101/256 and PASCAL VOC 2007/2011 datasets. a) Caltech101. b) Caltech-256. c) PASCAL VOC 2007/2011.

Some examples from Caltech101, Caltech256 and PASCAL VOC 2007/2011 are shown in Fig. 1. We can see that in Caltech101, most images are well aligned and basically without occlusion. We use Caltech101 because there are many algorithms that have been evaluated on it. Caltech256 is more challenging than Caltech101 due the large number of object classes, more diverse poses, background clutter and sizes. Compared to Caltech101/256, each image in the PASCAL VOC 2007 dataset may contain multiple labels. For example, “person” is the most common concept, appearing alongside many other concepts such as “dog”, “horse”, “bottle”, “chair” and “boat”, etc.

#### Caltech 101 and Caltech 256 datasets

In the Caltech101 dataset, there are 9144 images with 101 categories plus a ‘background’ category. The number of images in each category ranges from 31 to 800, and the image size is about 300×200 pixels. All 102 categories are used in the experiments. In the Caltech-256 dataset, there are 30,607 images from 256 categories plus a ‘background’ category. The number of images in each category ranges from 80 to 800.

#### PASCAL VOC 2007 dataset

The PASCAL-VOC 2007 dataset [39] consists of 9963 images from 20 classes. These images include indoor and outdoor scenes, close-ups and landscapes, and strange viewpoints. The dataset is divided into three parts: (i) a training set of 2501 images, (ii) a validation set of 2510 images and (iii) a test set comprising 4952 images.

#### PASCAL VOC 2011 dataset

We also conduct evaluation experiments on PASCAL-VOC 2011 [40], which consists of 14,961 images from 20 classes. Following the standard experiment setup for VOC 2011, we use 5717 images for training and 5823 images for testing. In general, the VOC datasets are challenging because the images are daily photographs that have been obtained from Flickr, with varying sizes, resolutions, viewing angles, illumination, appearances of objects, poses and occlusions.

#### 15-Scenes datasets

**Figure 2:**
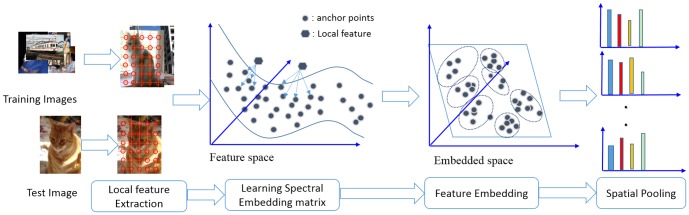
Mean accuracy of each category on 15-Scenes dataset.

In the 15-Scenes dataset, there are 4485 images with 15 categories, which are taken from the COREL collection, personal photographs and Google image search. The number of images in each category ranges from 200 to 400. The average image size is about 300×250. Some examples of each category are shown in Fig. 2.

### Experiment Settings

**Figure 3:**
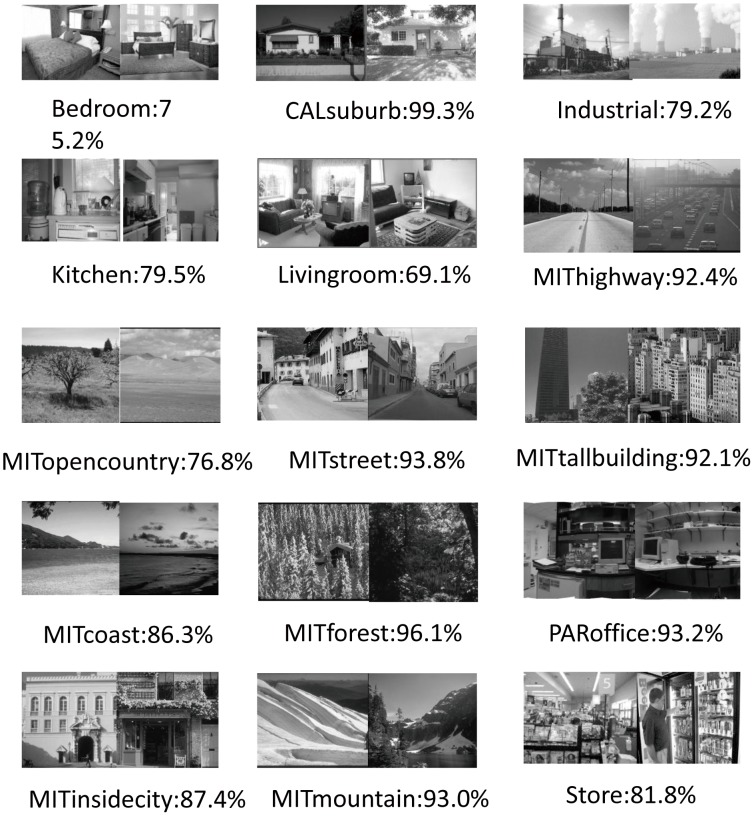
Image representation using local coding based spectral embedding.

As shown in [8], [19], [20], the image classification framework generally consists of (i) local feature extraction, (ii) feature quantization, (iii) spatial pooling and (iv) classifier learning stages We follow this framework except that we replace stage (ii) with feature embedding using the proposed LCMK method, as shown in Fig. 3.

In our experimental setting, images are first resized to keep the maximum size less than or equal to 300 pixels for the Caltech101/256 data set, while for PASCAL VOC 2007, the maximum size is set to 500 pixels. For local feature extraction, dense SIFT features are extracted on patches with three scales, i.e. 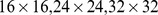, with step-size 4 for Caltech101/256,and step-size 2 for PASCAL VOC.

In feature embedding, a set of 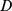 anchor points is obtained by applying *k*-means clustering on a 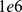 sized randomly selected training set. Following [8], we set 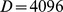 for the Caltech101/256 dataset, and 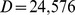 for PASCAL VOC. The embedding matrix 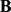 is learned off-line on the training set.

To incorporate spatial layout information, the linear version of spatial pyramid matching kernel [13], [15] is used, which adopts three levels of 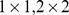 and 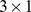 spatial divisions to introduce the rough spatial correspondence. The max-pooling operator is applied on embedded features belonging to each spatial division. The image is finally represented as the concatenated vector of each spatial division.

In classifier learning, the libsvm toolbox [49] is used to train the classifier for image classification. For the Caltech101/256 dataset, to keep consistency with the existing methods, we randomly split the image dataset into 5 pairs of training/test subset and report the mean classification accuracy.

### Experimental results

#### Experiment results on 15-scenes dataset

For the 15-Scenes dataset, 100 images per category are randomly selected as the training set, with the remainder selected as the test set. Furthermore, the training images are repeated with left-to-right mirroring to increase the size of the training set.

We learn the embedding matrix based on the 8192 anchor points trained on the randomly selected SIFT features. The performance is slightly better than the one learned with 4096 anchor points, which has performance: mAP(stdv) = 86.2±0.2%. The mean average precision (mAP) of 5 rounds of classification result is shown in Table 2. The classification accuracy of each category is shown in Fig. 2.

**Table 2:** Image classification results using 15-Scenes dataset in terms of mAP and stdv(%).

Method	Result
BoV [19]	81.40(0.39)
Sparse Coding [15]	80.3(0.5)
Macrofeature [20]	85.6(0.2)
LLC [13]	81.6(-)
LLC+ [41]	84.21
LCMK	**86.3(0.3)**

We can see that, by learning the embedding with our proposed LCMK method, the mAP result has been significantly improved, compared to LLC [13] and sparse coding [15]. The reason is perhaps that sparse coding mainly focuses on representing the local features with several visual words in the dictionary to reduce quantization error. LLC methods exploit the manifold structure in the original feature space, which shows a certain superiority over the sparse coding. To the best of our knowledge, the highest current performance for the Scenes-15 dataset using SIFT features is reported to be mAP(stdv) = 89.75±0.5% using the Laplacian sparse coding method [44]. Laplacian sparse coding considers the dependence of the sparse codes at the expense of efficiency. A computationally complex iterative optimization procedure is needed to construct the visual codebook and feature quantization.

In our proposed LCMK, we learn the embedding function from the Hilbert space derived from the local kernel matrix, which may exploit the manifold structure better. The performance of Macrofeatures [20] and LLC+ [41] is close to our results. In the Macrofeature method, discriminative training of the codebook is performed. LLC+ uses a similar idea to the Fisher Vector [18], which uses an image-dependent codebook derivative to represent the image, which is a high-dimensional representation.

#### Experiment results on Caltech 101 and Caltech 256 datasets

In this experiment, we first investigate the performance of LCMK for visual object classification on the Caltech101 dataset. Following the standard experimental settings, we train classifiers on 30 images, and test on no more than 50 images per category. A set of 4096 anchor points is used to learn the embedding matrix. We conduct 10 rounds of evaluation, and report the performance in Table 3.

**Table 3:** Image classification results using Caltech101 dataset in terms of mAP and stdv(%).

Method	Result
BoV [19]	76.95(0.39)
NBNN [10]	73.0(-)
Sparse Coding [15]	73.2(0.5)
LLC [13]	73.4(-)
O2P [35]	79.3(0.5)
Fisher Vector [18]	77.8(0.6)
HMP [50]	76.8(-)
LCMK	**80.2(0.4)**

From this, we can see that our LCMK method outperforms most of the listed algorithms, including Fisher Vector [18], and O2P [35]. The Fisher Vector exploits the first- and second-order statistics of the local features within a spatial region for better image representation. O2P leverages recent advances in computational differential geometry, which takes advantage of the Riemannian structure of the space of the symmetric positive definite matrices to summarize sets of local features inside regions. The performance of Fisher Vector and O2P show that appropriately pooling the sets of local features can significantly improve performance. Our proposed LCMK method could be easily combined with the Fisher Vector and O2P methods, since feature embedding is just a front-end processing of the local features. We leave this as our future work.

To further evaluate the scalability of the proposed LCMK method, with respect to more image categories and more images, we perform evaluations on the Caltech256 dataset with similar experimental settings as those used for Caltech101. We report the performance over 5 random trials in Table 4, with increasing training images selected per category. As shown, the performance of LCMK is consistently superior to the other listed algorithms, including Sparse Coding [15], LScSPM [44], Super Vector [16], LLC [13], LLC+ [41], O2P [35], Fisher Vector [18] and HMP [50].

**Table 4:** Image classification results using Caltech256 dataset in terms of mAP and stdv(%).

method/Training Images	15	30	45	60
BoV [38]	28.30	34.10	-	-
NBNN [10]	-	42.7(-)	-	-
Sparse Coding [15]	27.73	34.02	37.46	40.14
LScSPM [44]	30.0	35.74	38.54	40.43
Super Vector [16]	36.72	43.77	47.24	50.98
LLC [13]	34.36	41.19	45.31	47.68
LLC+ [41]	35.2(-)	42.8(-)	47.5(-)	51.2(-)
O2P [35] ^1^	-	42.6(0.4)	-	-
Fisher Vector [18]	38.5(0.2)	47.4(0.1)	52.1(0.4)	54.8(0.4)
HMP [50]	40.5(0.4)	48.0(0.2)	51.9(0.2)	55.2(0.3)
LCMK	**43.3(0.3)**	**51.5(0.2)**	**54.4(0.5)**	**57.9(0.4)**

#### Experiment results on PASCAL VOC 2007 and 2011 datasets

We evaluate our LCMK approach on the more challenging PASCAL VOC 2007 and 2011 datasets. For the VOC 2007 evaluation, we simply use the union of original training and validation divisions as the training set for classifier learning. The classification accuracy is measured using Average Precision (AP) based on the precision/recall curve. To maintain consistency with other reported results, we use the PASCAL toolkit to evaluate our proposed method. We refer to the detailed experiment results reported in [8]. That is, we learn feature embedding using 24,576 anchor points from 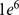 SIFT features sampled with step size 2. We also tried learning feature embedding using 4096 anchor points, yielding an AP of about 56.1%, worse than the figure we achieve. A possible explanation is that the latent manifold structure of visual objects with diverse sizes may not be effectively found by learning the embedding function from the randomly selected training features. Increasing the size of anchor points may improve the performance.

The experimental results are shown in Table 5. We can see that the proposed LCMK method outperforms LLC [13] as well as the winner of the PASCAL VOC 2007 [39]. The highest performance, with AP = 64.0%, was achieved by the Super Vector Coding method [16]. However this is achieved by applying several non-trivial modifications such as using LDA to compute an SVM kernel, and exploit second-order information as does the Fisher Vector [8]. Without these modifications, the performance of Super Vector coding is about AP = 58.2%, which is inferior to ours.

**Table 5:** Image classification results using PASCAL-VOC07 dataset in terms of mAP(%).

method/object class	aero	bicyc	bird	boat	bott	bus	car	cat	chair	cow
Winner of VOC07 [39]	77.5	63.6	56.1	71.9	33.1	60.6	78.0	58.8	53.5	42.6
LLC+ [41]	78.0	70.9	55.3	72.1	31.5	69.2	80.9	62.8	55.3	51.4
LLC [13]	74.1	64.9	51.5	68.3	27.2	62.9	78.4	61.4	54.4	47.2
Fisher Vector [18]	79.0	67.4	51.9	70.9	30.8	72.2	79.9	61.4	56.0	49.6
Super Vector [16]	79.4	72.5	55.6	73.8	34.0	72.4	83.4	63.6	56.6	52.8
LCMK	76.9	66.1	55.6	70.2	40.6	66.3	77.2	62.2	77.2	48.7

method/object class	table	dog	horse	m-bike	person	plant	sheep	sofa	train	tv	MAP
Winner of VOC07 [39]	77.5	63.6	56.1	71.9	33.1	60.6	78.0	58.8	53.5	42.6	59.4
LLC+ [41]	57.0	47.9	80.2	69.6	84.7	31.7	46.1	54.6	80.2	54.3	61.7
LLC [13]	52.8	44.6	78.1	68.5	83.7	29.9	51.0	55.5	78.6	53.6	59.3
Fisher Vector [18]	58.4	44.8	78.8	70.8	85.0	31.7	51.0	56.4	80.2	57.5	61.7
Super Vector [16]	63.2	49.5	80.9	71.9	85.1	36.4	46.5	59.8	83.3	58.9	64.0
LCMK	53.6	45.4	78.6	66.0	83.9	29.7	49.4	56.3	77.4	53.7	60.2

To further validate the efficiency and effectiveness of the proposed LCMK method, we also conduct the evaluation using the PASCAL VOC 2011 dataset. We report the experimental results of our proposed LCMK method with different codebook sizes, *i.e.* 4096, 8192,16,384 and 24,576, shown in Table 6. The best MAP we achieved is about 52.8%, outperforming results reported in [51] with the same experiment setup.

**Table 6:** Image classification results using PASCAL-VOC2011 dataset in terms of mAP(%).

codebook size/object class	aero	bicyc	bird	boat	bott	bus	car	cat	chair	cow
4096	75.9	46.4	40.6	50.0	18.7	74.1	52.2	55.1	45.8	21.9
8192	77.8	49.6	44.8	53.8	19.5	76.4	55.0	58.1	48.6	26.6
16384	78.6	51.3	48.1	55.8	22.6	77.2	56.6	61.4	51.8	26.7
24576	78.3	51.3	50.4	58.0	24.7	77.9	58.2	61.3	52.1	26.5

codebook size/object class	table	dog	horse	m-bike	person	plant	sheep	sofa	train	tv	MAP
4096	39.1	43.3	43.4	58.2	71.1	20.7	44.4	37.1	65.1	47.3	47.5
8192	39.2	45.0	43.7	58.1	74.1	17.5	45.0	42.1	66.4	53.6	49.8
16384	42.2	47.2	48.1	60.8	76.2	19.7	45.8	43.2	68.5	55.3	51.8
24576	43.0	47.6	48.2	60.4	77.0	24.2	46.8	43.9	70.5	55.8	52.8

## Conclusion and Future Work

This paper first presented a unified definition of visual matching for local feature based representation. The existing BoV and kernel based methods were then reviewed from a visual matching point of view, showing that local kernels defined over feature pairs plays an important role.

Since local features such as SIFT and HoG generally follow a heavy-tailed distribution, general Euclidean based local kernels may therefore yield poor matching accuracy and have undesirable side-effects. To address this issue, we proposed a local coding based matching kernel based method, termed LCMK, to exploit the manifold structure in the Hilbert space derived from the local kernel matrix. LCMK further combines advantages of both BoV and kernel based methods, and a linear computational complexity can be achieved. LCMK can therefore perform efficient and effective visual matching on large scale datasets. An evaluation conducted on image classification tasks using standard data sets reveals the superiority of the proposed LCMK method. However, especially for image classification on the more challenging PASCAL VOC 2007 dataset, there appears to still be potential to further improve performance, such as by exploiting second order information, using spectral embedding methods etc.

We anticipate that our future work will include: (i) Conducting experiments on more challenging large-scale ImageNet datasets to further validate the generalization capability of the LCMK method, (ii) To potentially incorporate the spatial pooling explored in Fisher Vector and O2P methods, which can exploit second order statistics, and (iii) to apply the proposed visual similarity measure to image retrieval tasks.
